# Clinical Outcomes and Complications of Bilateral Cosmetic Femoral Lengthening Using the Lengthening over Nail Technique: A Single-Center Retrospective Study

**DOI:** 10.3390/jcm15218321

**Published:** 2026-10-26

**Authors:** Yunus Oc, Muharrem Kanar, Esref Gursel, Emrah Kovalak

**Affiliations:** 1 Department of Orthopedics and Traumatology, Nisantasi Universtiy, Istanbul 34398, Turkey; 2 Department of Orthopedics and Traumatology, Sisli Hamidiye Etfal Training and Research Hospital, Health Sciences University, Istanbul 34371, Turkey; 3 Department of Orthopedics and Traumatology, Beykent University, Istanbul 34500, Turkey

**Keywords:** LON technique, cosmetic limb lengthening, lengthening over nail, femoral lengthening, distraction osteogenesis

## Abstract

**Background:** The primary objective of this study is to evaluate the clinical experience, efficacy, and limitations of the Lengthening Over Nail (LON) technique in cosmetic bilateral femoral lengthening. **Methods:** A retrospective analysis was conducted on 48 patients (44 males, 4 females) with constitutional short stature who underwent surgery between January 2022 and December 2023. Demographic data, preoperative height, lengthening ratio, time to full weight-bearing, and complications were evaluated. The association between variables was analyzed using Spearman’s rank correlation and Chi-square tests. **Results:** The mean age of the participants was 31.5 ± 9.43 years. A statistically significant weak negative correlation was observed between age and the amount of lengthening achieved (*p* = 0.038) as well as the lengthening ratio (*p* = 0.037). No significant difference was found in the mean age or lengthening ratio between patients with and without complications. The relationship between time to full weight-bearing and complications showed a trend toward significance (*p* = 0.068). **Discussion:** The LON technique is a sophisticated and reliable approach for aesthetic limb lengthening that integrates internal and external fixation to enhance stability and reduce external fixation duration **Conclusions**: While increasing age may be associated with slightly reduced lengthening outcomes, the procedure demonstrates acceptable complication rates when combined with careful patient selection and rigorous rehabilitation.

## 1. Introduction

In recent decades, limb-lengthening procedures have expanded beyond treating skeletal deformities caused by dwarfism, congenital anomalies, trauma, tumors, or infections, and are now increasingly performed for cosmetic purposes [1]. Cosmetic height enhancement is a surgical intervention that applies principles and techniques originally developed to address limb length discrepancies and deformities, aiming to increase an individual’s stature for aesthetic reasons [2]. The Ilizarov method, a groundbreaking approach involving gradual distraction osteogenesis, laid the foundation for modern limb-lengthening protocols. This process stimulates new bone formation by creating a corticotomy (surgical bone incision) and gradually separating bone segments, promoting regeneration within the osteotomy gap [3].

Early techniques for height-lengthening surgery were largely abandoned due to high complication rates [4,5]. Subsequent advancements led to modified surgical approaches based on Ilizarov’s distraction osteogenesis framework [6]. Among these, the lengthening over nail (LON) technique gained popularity by combining external fixation with an internal conventional intramedullary nail during the distraction phase. This hybrid method reduces the duration of external fixation compared to traditional Ilizarov methods and enhances stability through intramedullary support [7]. The LON technique combines the benefits of external fixation and intramedullary stabilization, providing mechanical support during consolidation while potentially reducing the duration of external fixator use [2,3]. Despite these advantages, debates persist regarding its efficacy in bone consolidation [8,9].

While the LON method employs an intramedullary nail to internally stabilize newly formed bone and an external fixator to control lengthening speed and alignment, its impact on callus regeneration during distraction osteogenesis remains unclear. Studies report conflicting outcomes: some found no significant difference in healing index (treatment duration per unit of bone regeneration) between LON and classical Ilizarov methods [10,11], while others noted a lower healing index with LON [12,13]. Critically, all these studies used healing index as the endpoint, which fails to quantitatively assess bone regeneration during the active distraction phase.

The integration of intramedullary nails with external fixation presents a promising strategy for shortening external fixation duration and enhancing mechanical stability throughout bone consolidation. Emerging as a preferred option in cosmetic height enhancement, the LON technique has gained traction due to its potential to optimize outcomes while mitigating complications. Initially conceptualized to address limb length discrepancies, the principles of distraction osteogenesis have now been strategically adapted to meet the demands of aesthetic surgery. The ongoing refinement of surgical methods, including LON-based lengthening, underscores the critical need for innovation in maximizing patient success and minimizing procedural risks.

Nevertheless, the literature remains sparse regarding the application of the LON method for cosmetic height augmentation, with limited data on its inherent risks and long-term benefits. This gap highlights an understudied area in elective orthopedic interventions.

The primary objective of this study was to evaluate the clinical and radiographic outcomes of bilateral cosmetic femoral lengthening using the LON technique, with particular emphasis on the magnitude of achieved lengthening, postoperative complications, and functional recovery milestones. A secondary objective was to investigate associations between patient characteristics and clinical outcomes, while acknowledging the potential influence of preoperative treatment goals and other confounding factors.

## 2. Materials and Methods

This single-center retrospective study was conducted in accordance with the Declaration of Helsinki and was approved by the Ethics Committee of Şişli Hamidiye Etfal Training and Research Hospital (approval number: 2025/4844). Clinical trial registration was not applicable to this retrospective observational study.

### 2.1. Study Design and Patient Selection

This single-center retrospective study included 48 patients (44 men and four women), representing 96 femora, who underwent bilateral elective cosmetic femoral lengthening using the LON technique between January 2022 and December 2023. All procedures were performed for aesthetic stature augmentation rather than correction of a documented pathological limb-length discrepancy or skeletal deformity.

Preoperative assessment was reviewed to identify potential pathological causes of short stature, including skeletal dysplasia, endocrine disorders, genetic conditions, and previous orthopedic procedures involving the lower extremities. The presence or absence of these conditions was determined from the available medical history, clinical examination findings, and relevant investigations documented before surgery.

Patient-level data included age, sex, preoperative and postoperative standing height, the recorded amount of lengthening achieved, time to full weight-bearing, and postoperative complications. Although bilateral femoral lengthening was performed in all patients, the available dataset contained a single achieved-lengthening value per patient rather than separate measurements for the right and left femora. The height-based lengthening ratio was calculated by dividing the recorded length gain by preoperative standing height and multiplying the result by 100. As baseline femoral segment lengths were unavailable, the percentage of elongation relative to the initial femoral length could not be determined.

Statistical analyses were originally performed using IBM SPSS Statistics for Windows, version 25.0 (IBM Corp., Armonk, NY, USA). Descriptive statistics were expressed as mean ± standard deviation and range for continuous variables and as frequencies and percentages for categorical variables. Spearman’s rank correlation coefficient was used to assess the associations between patient age and both achieved lengthening and the height-based lengthening ratio. Comparisons of continuous variables between patients with and without complications were performed using Welch’s independent-samples *t*-test. The association between time to full weight-bearing and complication status was explored using Pearson’s chi-square test. All analyses were conducted at the patient level (n = 48), without treating the bilateral femoral measurements as independent observations. Statistical significance was defined as *p* < 0.05. All patient-level statistical analyses were performed using the individual patient as the unit of observation. Given the bilateral nature of the procedures, measurements obtained from the right and left femora of the same patient were not treated as independent observations. Continuous variables were summarized using means, standard deviations, and ranges, whereas categorical variables were expressed as frequencies and percentages. A *p*-value of <0.05 was considered statistically significant.

This study was reported in accordance with the Strengthening the Reporting of Observational Studies in Epidemiology (STROBE) recommendations.

### 2.2. Surgical Technique and Patient Follow-Up

#### 2.2.1. Preoperative Planning

**Figure 1:**
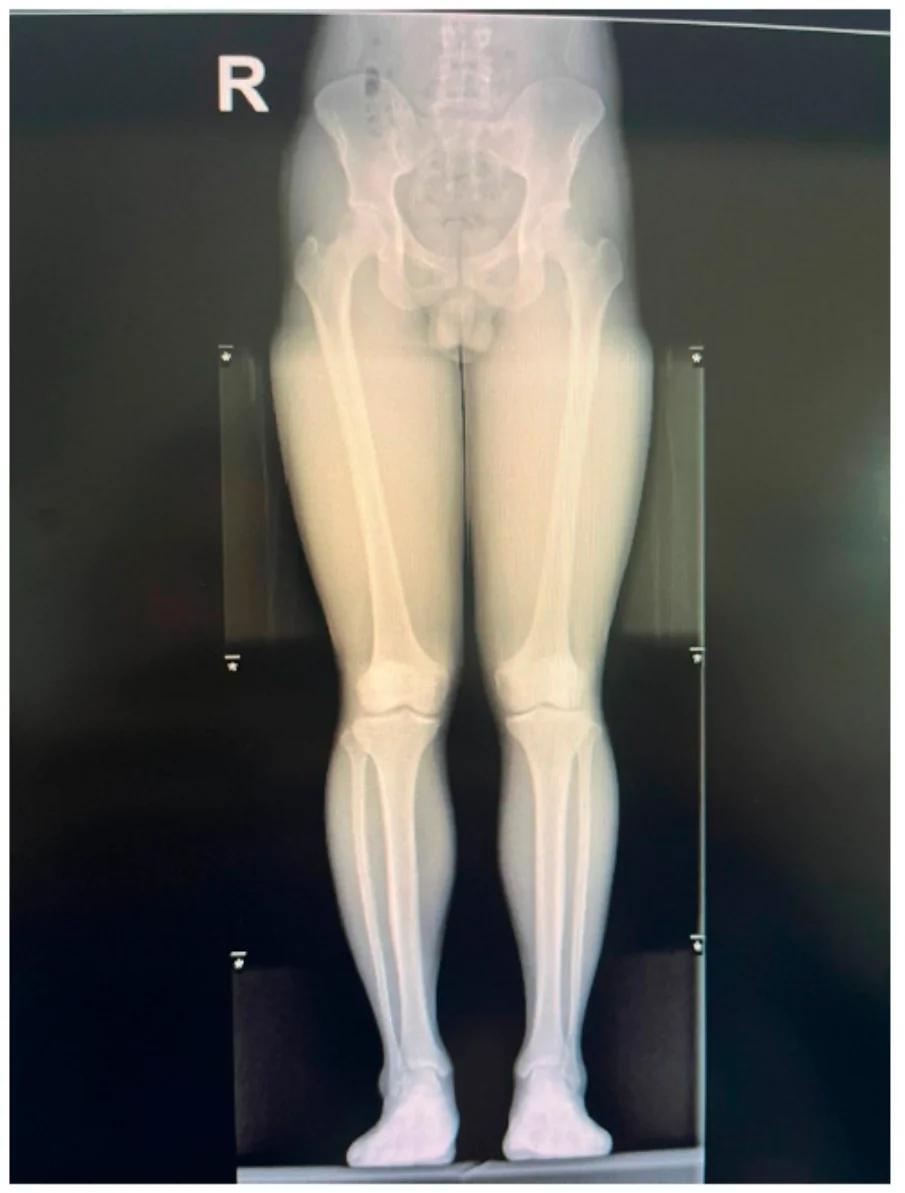
Preoperative long-leg standing orthoradiograph demonstrating the initial deformity analysis. This view was used to determine the level of osteotomy and to measure the appropriate length and diameter of the intramedullary nail.

Preoperative planning was based on full-length standing lower-extremity radiographs, which were used to assess lower-limb alignment, identify potential deformities, and determine the appropriate osteotomy level and intramedullary implant dimensions (Figure 1). Particular attention was given to the anticipated effects of isolated femoral lengthening on overall lower-extremity proportions. When calibrated segmental measurements were available, femoral and tibial lengths were assessed separately to evaluate the potential changes in the femur-to-tibia length relationship following the planned procedure.

#### 2.2.2. Surgical Procedure

All operations were performed under general anesthesia with the patient in the supine position. A radiolucent bump was placed under the hip of the operative side. In accordance with the preoperative plan, subtrochanteric drill holes were created to mark the osteotomy site, typically 5–7 cm distal to the lesser trochanter. A 2-cm incision was then made 7 cm proximal to the greater trochanter. The medullary canal was accessed via the piriform fossa and reamed. A conventional intramedullary nail (Tasarımmed; Istanbul, Türkiye) with a diameter 3 mm smaller than that of the final reamer was inserted into the femoral canal.

With the nail in situ, two Schanz screws were inserted at the level of the lesser trochanter. An external fixation system (Tasarımmed; Istanbul, Türkiye) was attached to the proximal Schanz pins to establish and maintain the planned distraction axis. To ensure the fixator axis correlated with the nail, a single distal Schanz screw was inserted, aligned with the distal portion of the nail. Subsequently, the external fixator and the intramedullary nail were temporarily extracted, and the osteotomy was completed through the pre-drilled site.

Following the osteotomy, the intramedullary nail was re-inserted and locked proximally with two screws. The external fixator was re-attached to the Schanz screws. A second distal Schanz screw was inserted with the knee in flexion. Prior to the placement of the final Schanz screw, a percutaneous iliotibial band release was performed at the level of the superior pole of the patella. Lengthening apparatuses were attached to the external fixator, and an acute 1-cm distraction test was performed intraoperatively to verify the completeness of the osteotomy. Upon confirmation of successful distraction, the wounds were irrigated and closed. The identical protocol was performed on the contralateral side during the same session.

#### 2.2.3. Postoperative Management

Perioperative antibiotic prophylaxis was administered in accordance with the institutional protocol. Patients received intravenous cefazolin (2 g, or 3 g for patients weighing ≥ 120 kg) within 60 min before surgical incision. Intraoperative redosing was performed when indicated by the duration of surgery, with an approximately 4-h redosing interval. Antibiotic prophylaxis was discontinued within 24 h postoperatively.

Postoperative rehabilitation was initiated during hospitalization. Assisted mobilization using a walker began on postoperative day 1, followed by a structured rehabilitation program starting on postoperative day 2. After discharge, patients were prescribed supervised physiotherapy sessions lasting approximately 2 h per day, at least five days per week. Rehabilitation was continued throughout the distraction and consolidation phases.

Following completion of the distraction phase, distal interlocking of the intramedullary nails and removal of the external fixators were performed. Patients were subsequently maintained on a non-weight-bearing mobilization protocol using a walker for approximately six weeks while continuing rehabilitation. Progression to partial weight-bearing with bilateral crutches was determined by clinical and radiographic assessment of regenerate formation. Unrestricted weight-bearing without assistive devices was permitted after satisfactory radiographic consolidation had been demonstrated.

The principal rehabilitation objectives were progression determined according to pain, joint mobility, muscular function, weight-bearing tolerance, and radiographic healing.

Patients were hospitalized for four days postoperatively. Ambulation with a walker was initiated on postoperative day 1, and rehabilitation commenced on day 2. The distraction protocol began on day 5, concurrent with hospital discharge.

Following discharge, patients underwent a rehabilitation regimen consisting of 2-h sessions, at least five days per week.

**Figure 2:**
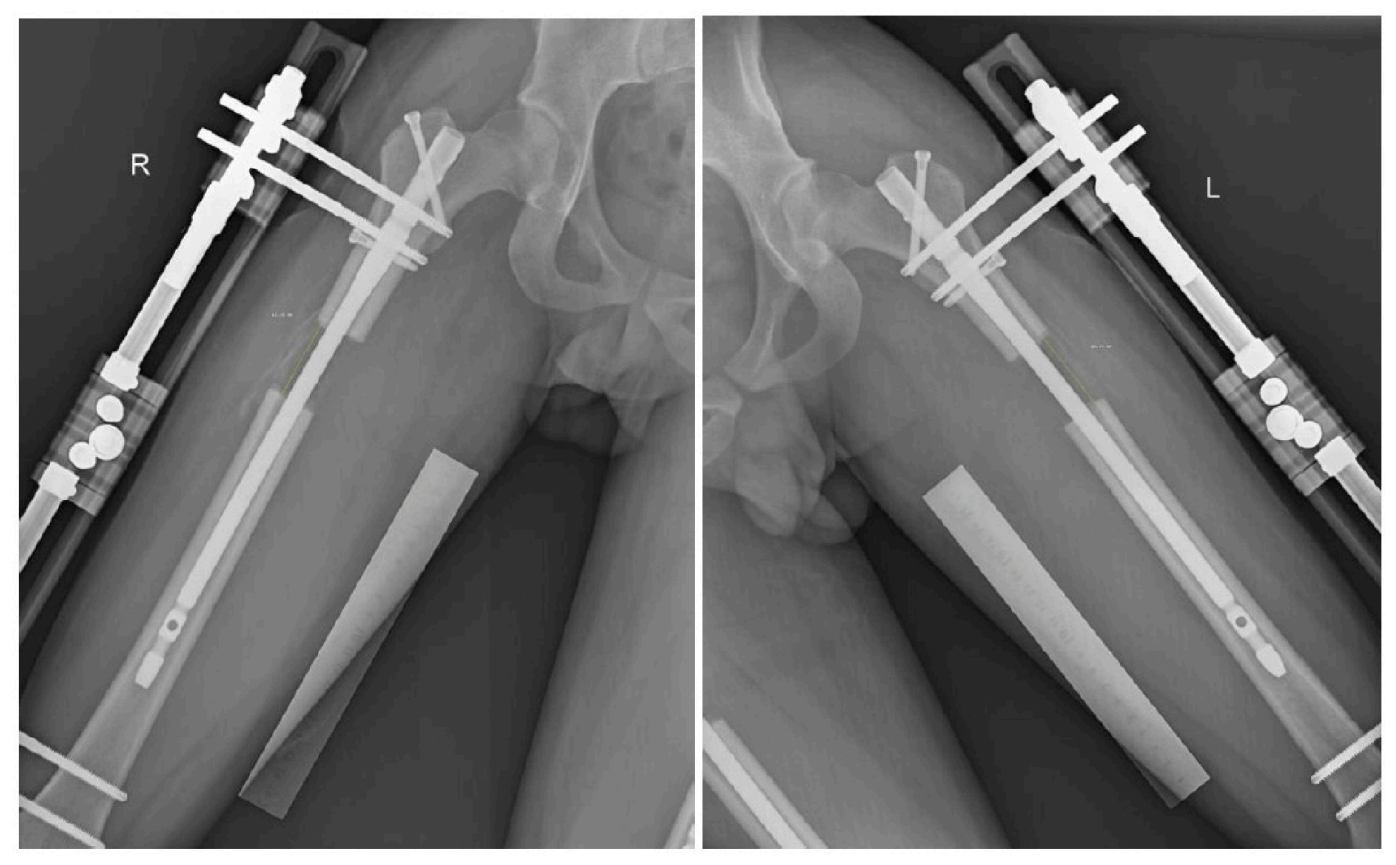
Follow-up radiograph obtained during the distraction phase using a portable X-ray unit. This image confirms the progression of lengthening and the alignment of the bone segments.

**Figure 3:**
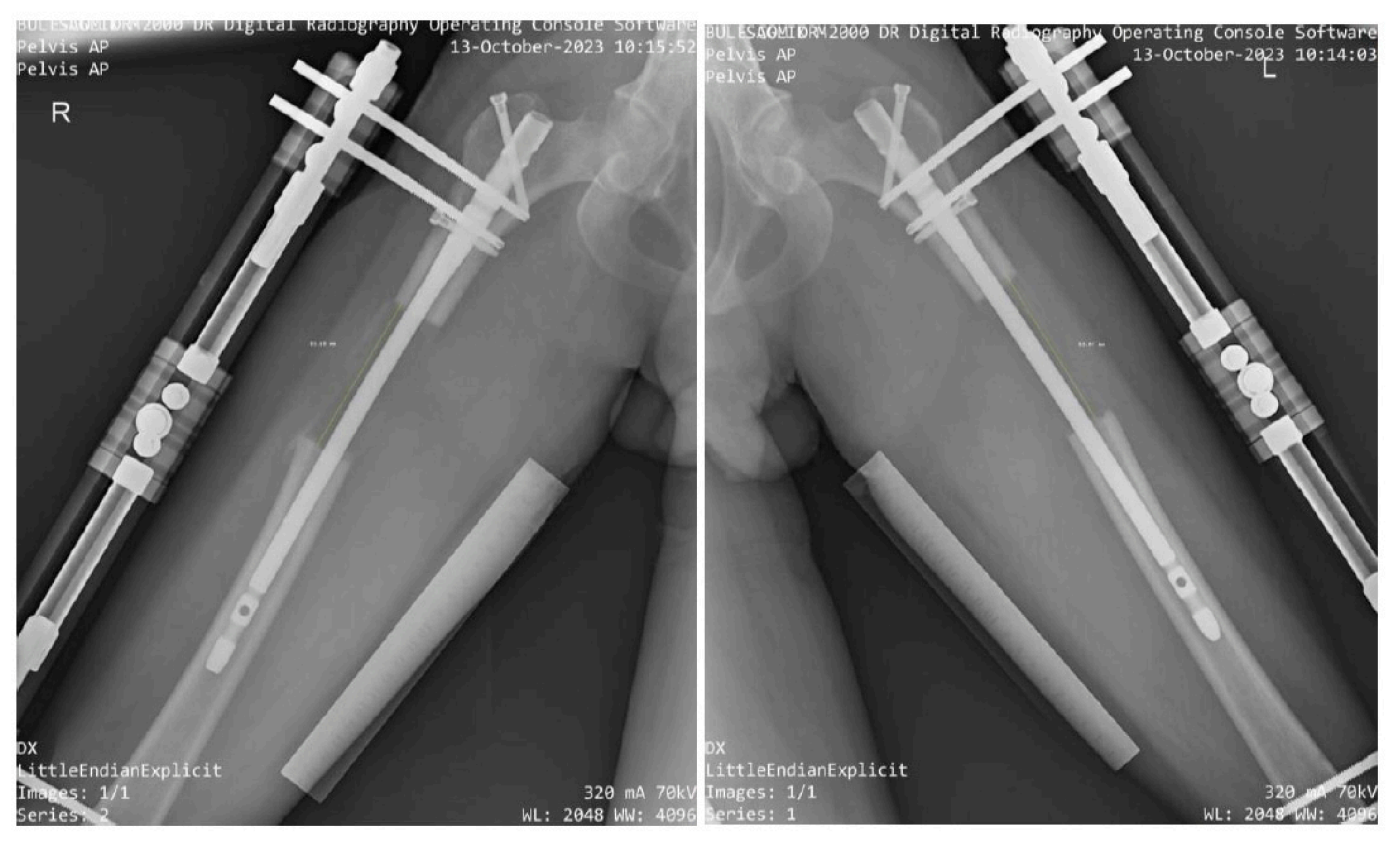
Radiograph taken at the completion of the distraction phase. The image shows that the target length has been achieved equally in both limbs and the regenerate quality is being monitored prior to the consolidation phase.

Gradual distraction was initiated on postoperative day 5 and performed as a single 1-mm adjustment each day. Serial radiographic examinations were performed at approximately 1, 3, 5, and 7 cm of distraction, when applicable, to evaluate bilateral lengthening symmetry, mechanical alignment, and regenerate bone formation (Figure 2 and Figure 3).

#### 2.2.4. Consolidation and Hardware Removal

Upon achieving the target length, patients were readmitted for the consolidation phase. Under general anesthesia, the intramedullary nails were locked distally with two screws, and the external fixators and Schanz screws were removed. The pin sites were debrided and dressed.

**Figure 4:**
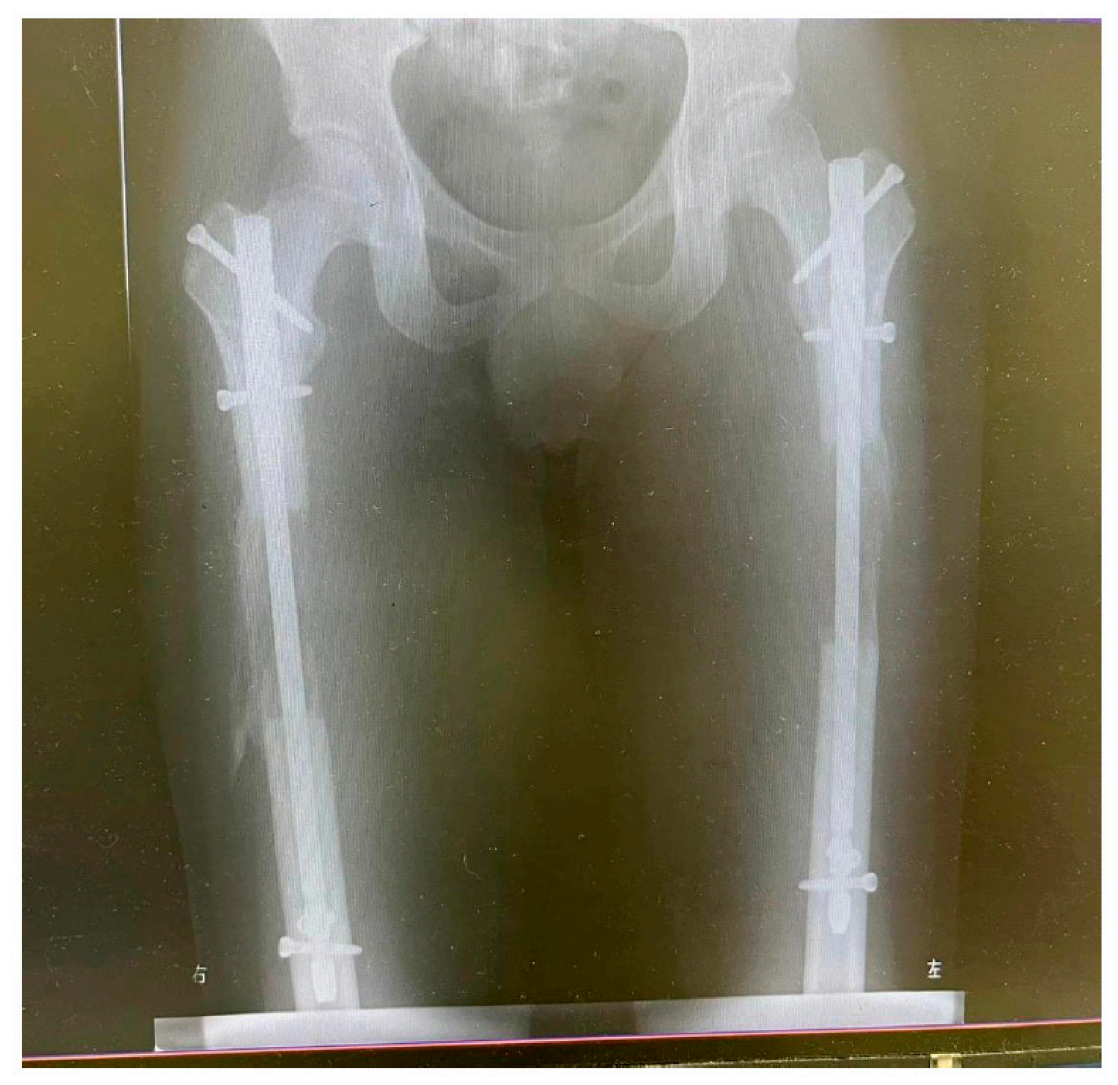
Anteroposterior radiograph obtained six weeks after the removal of the external fixator. The intramedullary nail is statically locked distally. Note the visible callus formation (regenerate) bridging the osteotomy site, which permitted the transition to partial weight-bearing with crutches.

**Figure 5:**
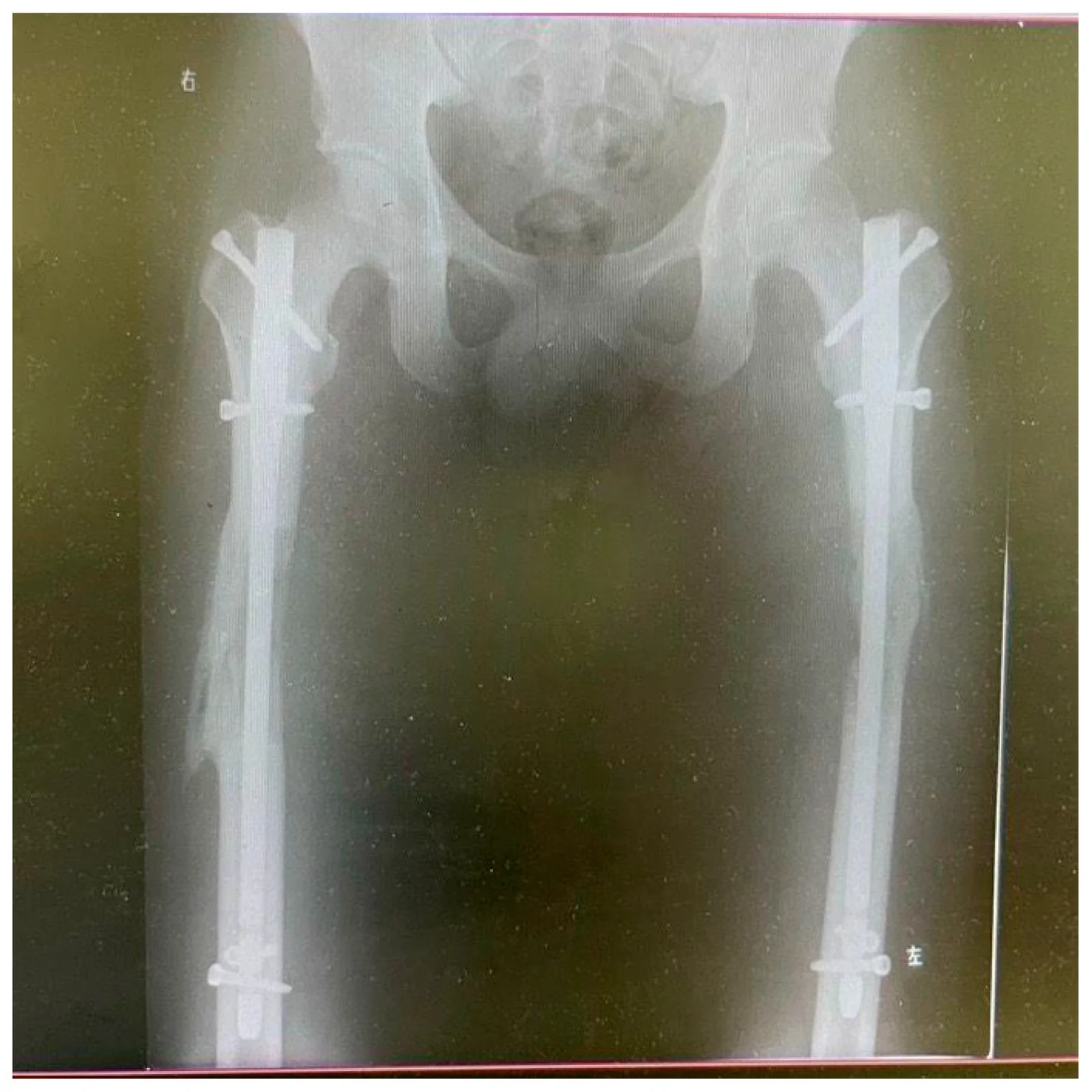
Final follow-up radiograph taken three months after external fixator removal. The regenerate demonstrates full consolidation and corticalization, indicating sufficient structural integrity for full weight-bearing ambulation without assistive devices.

**Figure 6:**
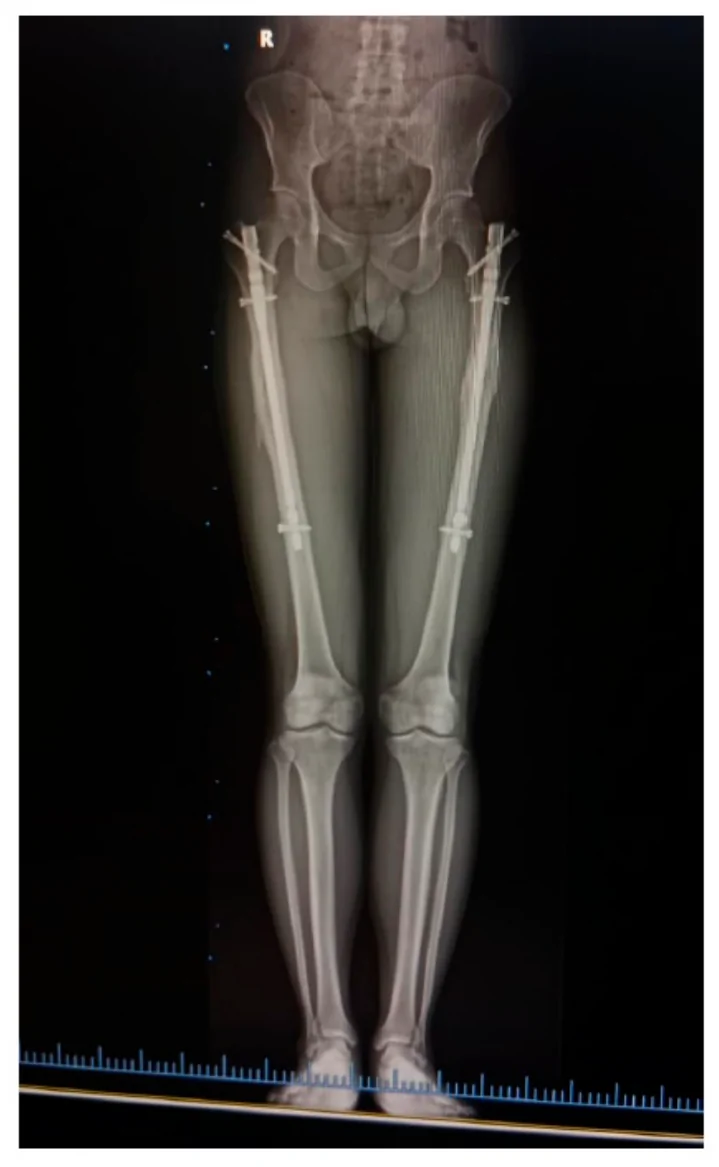
Long-leg standing radiograph obtained at 1.5 years postoperatively, immediately prior to the removal of the intramedullary nails. The image demonstrates the maintenance of limb length equality and complete consolidation.

Following the removal of the external fixator, patients were mobilized non-weight-bearing with a walker for six weeks while continuing rehabilitation. Control radiographs were obtained at six weeks; if sufficient callus formation was observed, patients were transitioned to partial weight-bearing using crutches (Figure 4). Full weight-bearing without assistive devices was permitted three months after fixator removal, contingent upon radiographic evaluation of the consolidation (Figure 5). Long-term follow-up continued to monitor the stability of the correction. At 1.5 years postoperatively, a final long-leg standing radiograph was obtained to confirm the maintenance of limb length equality and complete bone consolidation prior to the elective removal of the intramedullary nails (Figure 6).

Time to full weight-bearing was recorded as a postoperative recovery milestone. However, this parameter was not considered equivalent to complete functional recovery, as standardized measures of gait, muscle strength, return to work, and unrestricted daily activity were not systematically available.

#### 2.2.5. Radiographic Outcome Assessment

Radiographic healing was evaluated using serial anteroposterior and lateral femoral radiographs obtained during distraction and consolidation. The assessments focused on regenerate formation, cortical bridging, axial alignment, and progression toward consolidation. Where suitable serial radiographs were available, the time to radiographic consolidation and the number of bridged cortices were evaluated retrospectively.

The external fixation index was defined as the duration of external fixation in days divided by the achieved lengthening in centimeters. The consolidation index was defined as the interval from osteotomy to radiographically established consolidation, divided by the achieved lengthening. Patients with delayed union or nonunion were identified from the clinical and radiographic records.

## 3. Results

A total of 48 patients, representing 96 femora, underwent bilateral cosmetic femoral lengthening using the LON technique. The mean patient age was 31.50 ± 9.43 years (range, 19–62 years). The study population comprised 44 men (91.7%) and four women (8.3%). The mean preoperative standing height was 167.46 ± 6.05 cm (range, 150–181 cm). Baseline demographic and anthropometric characteristics are summarized in Table 1.

The mean achieved lengthening was 7.14 ± 1.24 cm (range, 5.0–9.5 cm), corresponding to a mean height-based lengthening ratio of 4.27 ± 0.73% (range, 2.86–5.57%). The mean postoperative standing height was 174.71 ± 6.20 cm (range, 156–187 cm). The mean time to full weight-bearing was 5.88 ± 0.82 months (range, 5–9 months). These findings demonstrate the magnitude and variability of lengthening achieved across the study population. Detailed lengthening and postoperative recovery outcomes are presented in Table 2.

**Figure 7:**
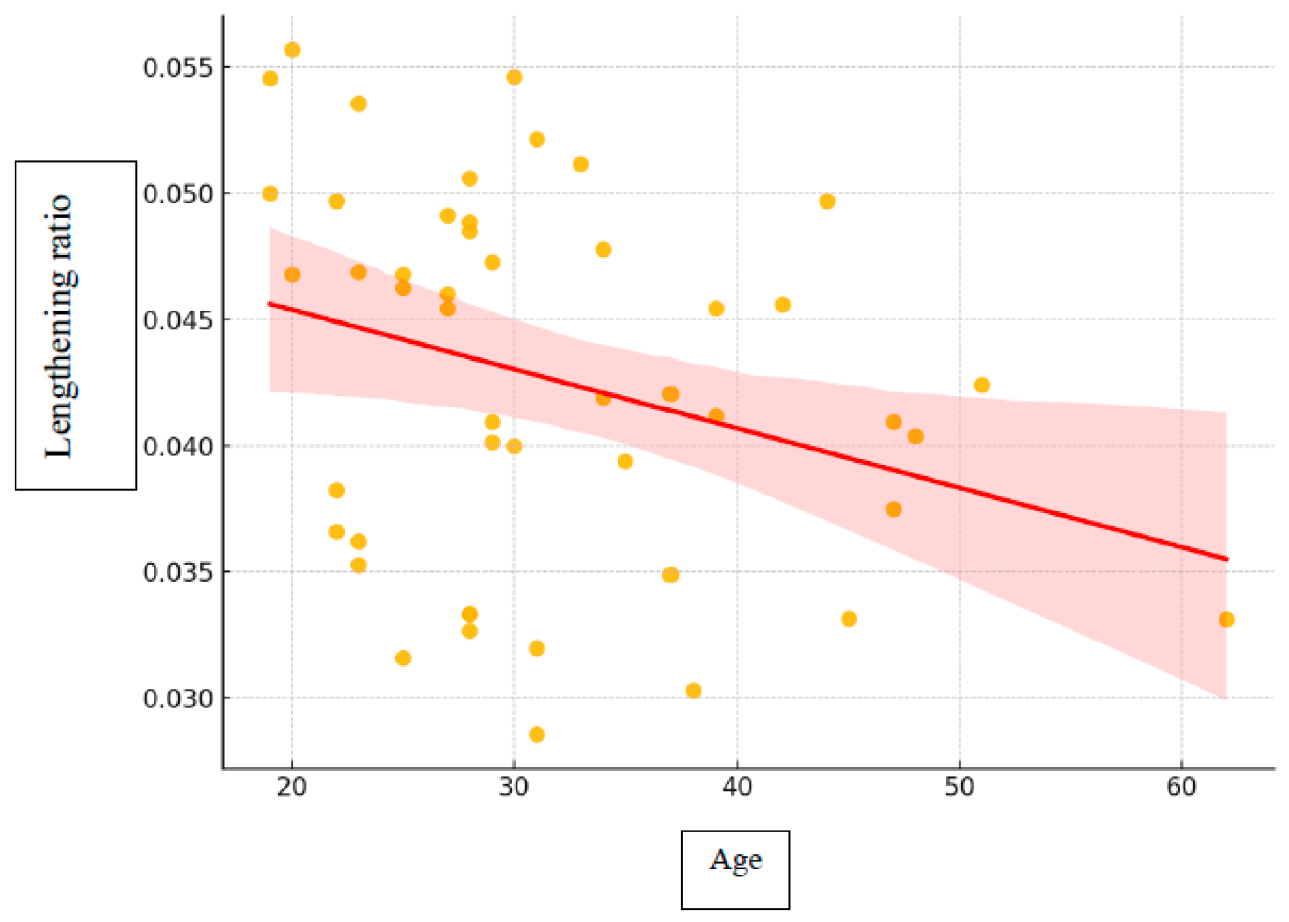
Scatter plot demonstrating the association between patient age and the height-based lengthening ratio. A statistically significant weak negative correlation was observed (Spearman’s r = −0.298, *p* = 0.039).

Spearman’s correlation analysis demonstrated a statistically significant weak negative association between patient age and the amount of lengthening achieved (r = −0.313, *p* = 0.030). Similarly, a weak negative correlation was observed between patient age and the height-based lengthening ratio (r = −0.298, *p* = 0.039), as illustrated in Figure 7.

**Figure 8:**
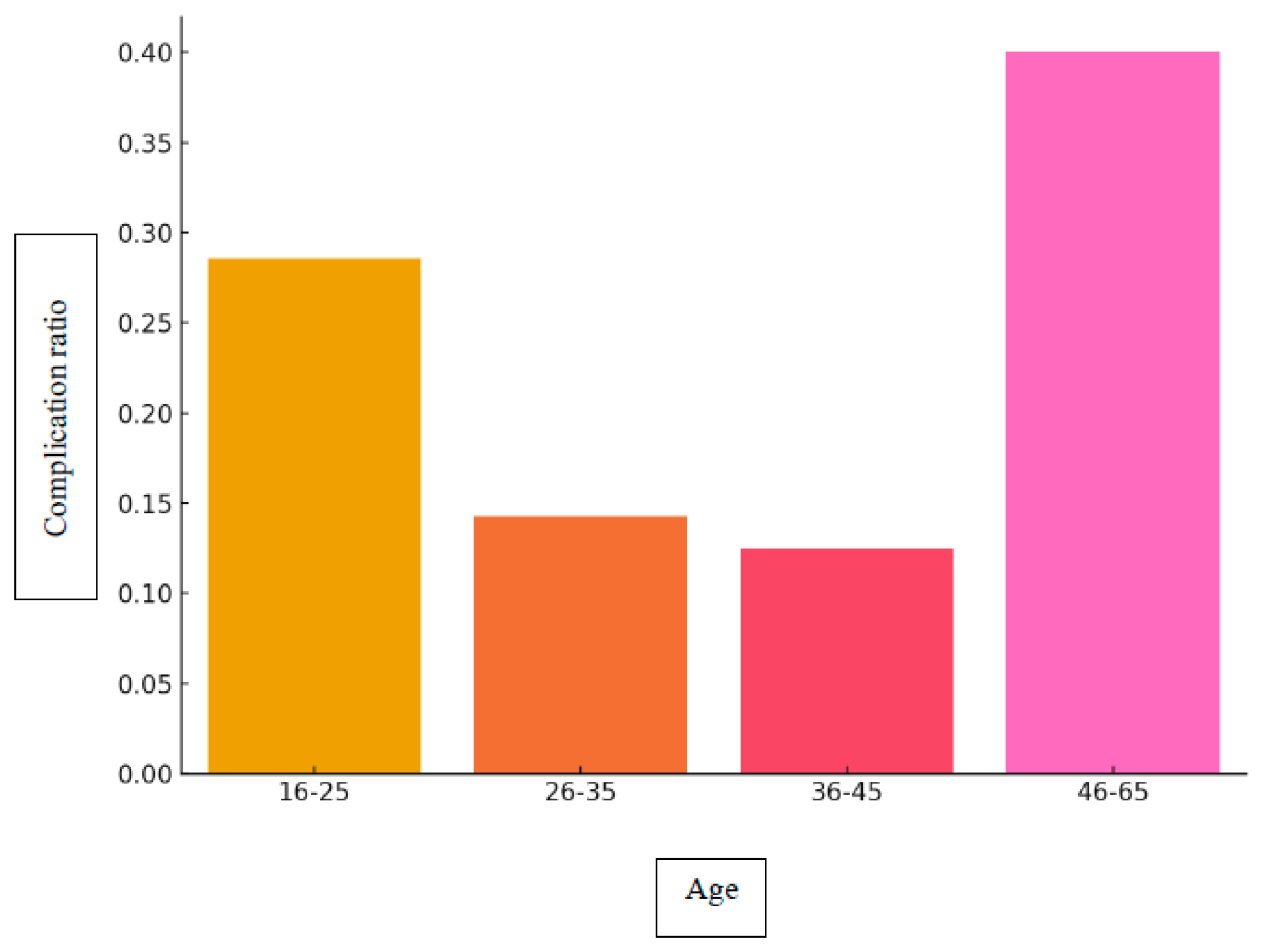
Distribution of complication ratios according to age groups. The analysis indicated that age was not a statistically significant predictive factor for the development of complications.

Postoperative complications were documented in 10 of the 48 patients (20.8%), whereas 38 patients (79.2%) had no recorded complications. The reported complications included pin-site infection (n = 2), neuropathy (n = 2), delayed union (n = 2), nonunion or pseudarthrosis (n = 2), Schanz pin-related revision following a fall (n = 1), and knee flexion restriction requiring quadricepsplasty (n = 1). Intramedullary nail breakage was additionally reported in one patient with nonunion. The mean age was 31.4 years among patients with complications and 31.5 years among those without complications, with no statistically significant between-group difference (*p* = 0.973; Figure 8).

**Figure 9:**
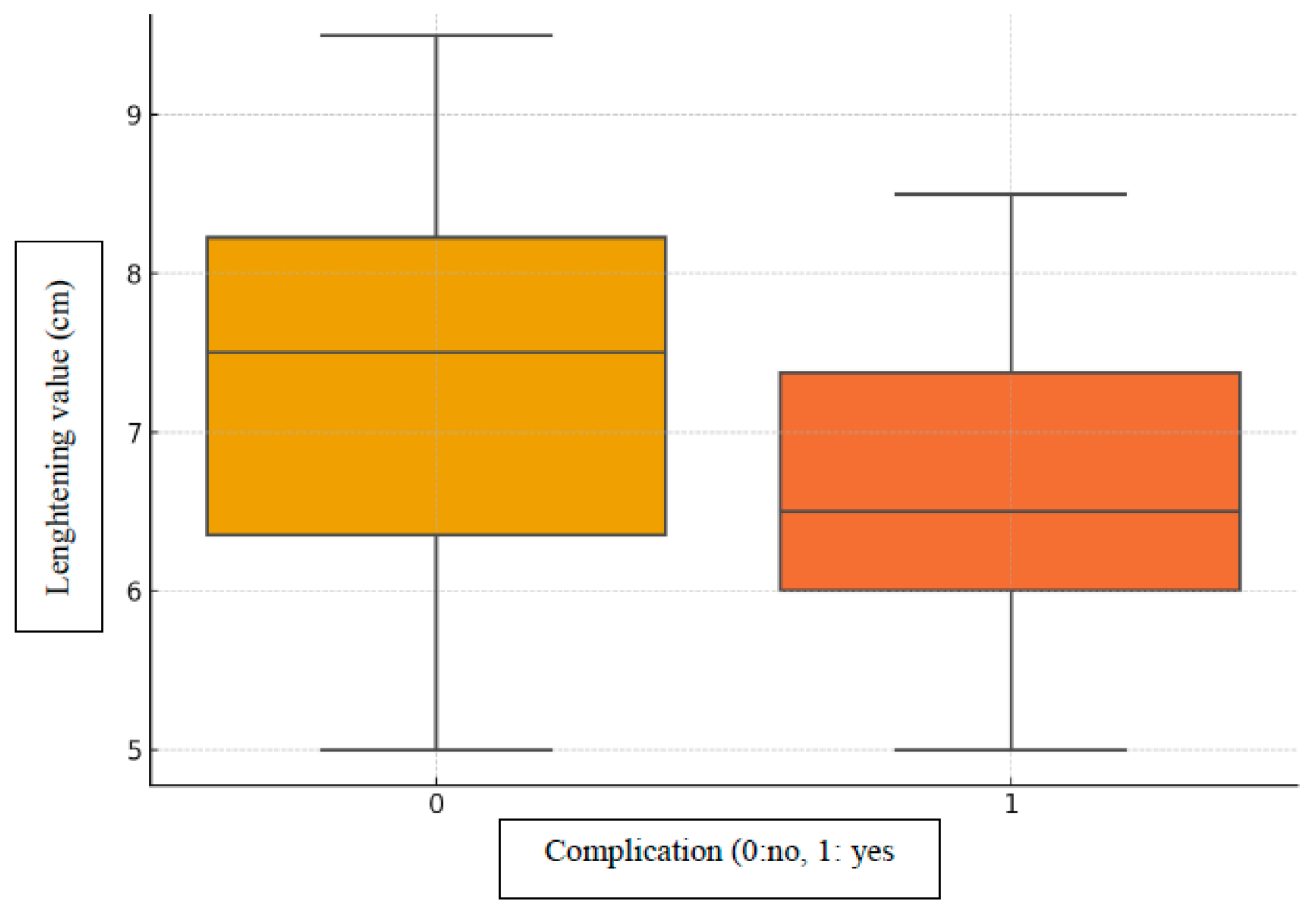
Distribution of lengthening values according to complication status. Statistical analysis revealed that the amount of lengthening did not differ significantly between patients who developed complications and those who did not (*p* = 0.240).

The mean achieved lengthening was 6.68 ± 1.17 cm in patients who developed complications and 7.27 ± 1.24 cm in those without complications. The difference was not statistically significant (*p* = 0.186; Figure 9). Similarly, no statistically significant difference was identified in the height-based lengthening ratio between patients with and without complications (4.03 ± 0.67% vs. 4.33 ± 0.75%, respectively; *p* = 0.240).

Furthermore, the impact of the lengthening magnitude on the complication rate was evaluated. The mean lengthening amount in patients with complications was found to be comparable to that of the complication-free group. This difference did not reach statistical significance (*p* = 0.240), indicating that the amount of lengthening was not a predictive factor for the development of complications (Figure 9).

**Figure 10:**
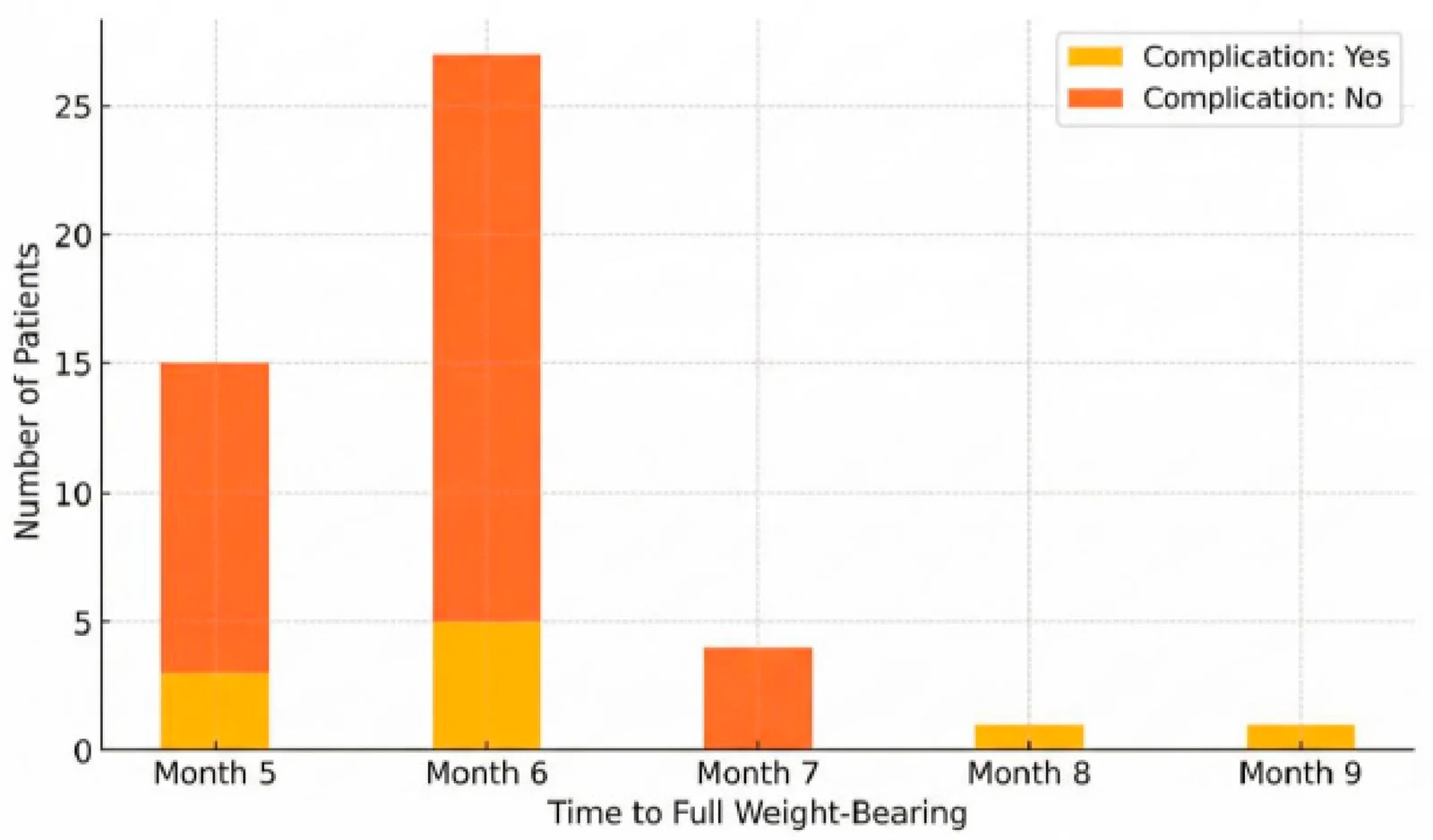
Frequency distribution of patients achieving full weight-bearing at different postoperative months. The analysis aimed to determine if complications delayed the time to full weight-bearing. Although a trend was observed, the relationship did not reach statistical significance (*p* = 0.068).

The mean time to full weight-bearing was 5.88 ± 0.82 months (range, 5–9 months). Full weight-bearing was achieved by 15 patients in the fifth postoperative month, 27 patients in the sixth month, four patients in the seventh month, and one patient each in the eighth and ninth months. The association between time to full weight-bearing and complication status did not reach statistical significance in the initial chi-square analysis (*p* = 0.068; Figure 10). However, this finding should be interpreted cautiously because of the small number of observations in some categories.

### Patient-Reported Pain Outcomes

At the 12-month assessment, pain intensity was documented using the Visual Analog Scale (VAS) in all 48 patients. The mean VAS score was 0.19 ± 0.67, with a median of 0 (range, 0–3). A VAS score of 0 was recorded in 44 patients (91.7%), whereas four patients (8.3%) reported residual pain scores between 1 and 3.

Although this value does not meet the threshold for statistical significance, it indicates a trend toward significance. It is possible that the limited sample size reduced the statistical power of this analysis.

## 4. Discussion

This retrospective single-center study evaluated the clinical and radiographic outcomes of bilateral cosmetic femoral lengthening using the LON technique in 48 patients with constitutional short stature. The principal findings were a mean achieved lengthening of 7.14 ± 1.24 cm, a mean height-based lengthening ratio of 4.27 ± 0.73%, and a postoperative complication rate of 20.8%. Furthermore, patient age demonstrated a weak negative correlation with both achieved lengthening and the height-based lengthening ratio. These findings provide quantitative insight into the clinical outcomes of LON-assisted cosmetic femoral lengthening, although the retrospective design and absence of a comparative group limit conclusions regarding its relative effectiveness and safety.

The LON technique combines the advantages of external fixation with the biomechanical stability of an intramedullary nail. As observed in our cohort, the external fixator provides precise control over the distraction rhythm, optimizing bone regeneration [2]. Concurrently, the intramedullary nail neutralizes bending and shear forces, preventing axial deviations and protecting the regenerate during the consolidation phase. This synergistic effect minimizes the duration of external fixator use—a critical factor in reducing fixator-related complications such as pin-site infections and soft tissue contractures.

From a biomechanical perspective, femoral geometry and loading conditions may influence structural behavior and stress distribution. Previous patient-specific finite element investigations have demonstrated that femora of different lengths can exhibit distinct mechanical responses under simulated loading conditions. Although these findings were not obtained in surgically lengthened femora, they highlight the relevance of femoral morphology and loading characteristics when considering postoperative rehabilitation and mechanical stability. Nevertheless, the present study did not directly assess implant stresses, patient-specific loading patterns, or finite element-derived mechanical parameters [13].

An additional consideration in isolated cosmetic femoral lengthening is the potential alteration of lower-extremity segmental proportions. Because femoral lengthening increases thigh length without a corresponding change in tibial length, the femur-to-tibia length relationship may be altered following surgery. Such changes could theoretically influence lower-extremity biomechanics, gait characteristics, and patient-perceived body proportions. However, standardized preoperative and postoperative segmental measurements, gait analyses, and validated body-image assessments were not available in the present cohort. Consequently, the clinical and psychological implications of postoperative proportional changes could not be determined.

A notable finding of the present study was the weak inverse correlation between patient age and the magnitude of lengthening achieved (r = −0.313, *p* = 0.030). A similar association was observed for the height-based lengthening ratio (r = −0.298, *p* = 0.039). Although these findings suggest that older patients tended to achieve smaller length gains, the retrospective design does not allow a causal relationship to be established. Factors such as individual lengthening targets, soft-tissue tolerance, and rehabilitation adherence may also influence the achieved lengthening magnitude.

In the present cohort, complications were documented in 20.8% of patients. Neither patient age nor the magnitude of achieved lengthening demonstrated a statistically significant association with complication occurrence. However, the relatively small number of patients experiencing complications limits the statistical power of these comparisons. Therefore, the absence of statistical significance should not be interpreted as evidence that age or lengthening magnitude has no influence on complication risk.

Reamed intramedullary nailing has become the standard treatment modality for diaphyseal and selected metaphyseal fractures of long bones [14,15]. Although early-phase studies have demonstrated that reaming can temporarily impair endosteal and cortical blood flow, it is also believed to positively influence bone healing through several biological mechanisms [15]. These mechanisms include compensatory increases in periosteal circulation [16], local graft-like effects of intramedullary debris, osteoinductive properties, and the activation of growth factors and inflammatory responses that promote bone healing [17,18]. However, the specific impact of intramedullary reaming on bone regeneration during distraction osteogenesis remains incompletely understood.

One study reported that blood flow in the lengthened limb increased tenfold by the second week post-distraction compared to the contralateral limb, and remained four to five times higher during the remainder of the distraction phase [19]. Another study demonstrated that osteogenic tissue filled the distraction gap through re-established vascular connections between the periosteal and intramedullary circulations [20].

Intramedullary reaming has been associated with biological responses that may influence bone healing, including compensatory changes in periosteal circulation and the potential osteogenic effects of reaming debris. However, the contribution of these mechanisms to regenerate formation during distraction osteogenesis remains incompletely understood. Importantly, the biological activity and potential osteoinductive properties of reaming debris were not directly investigated in the present study. Therefore, any proposed beneficial effect of intramedullary reaming on regenerate formation remains hypothetical and cannot be substantiated by our findings.

In our country, no form of cosmetic height-lengthening surgery is covered by public or private health insurance systems. This creates a significant barrier for individuals seeking surgical intervention for aesthetic height concerns. Compared to techniques involving motorized nails, the LON method remains a more cost-effective alternative.

Although cosmetic stature augmentation may influence patient satisfaction, perceived body image, and quality of life, standardized psychological assessments and validated patient-reported outcome measures were not systematically available in this retrospective cohort. Consequently, the present study cannot determine the effects of the intervention on self-confidence, psychosocial well-being, or overall patient satisfaction. The availability of postoperative pain VAS scores provides limited information regarding patient-reported pain but does not constitute a comprehensive assessment of functional or psychological outcomes.

Although the LON technique facilitated the achievement of the recorded lengthening outcomes, postoperative adverse events occurred in approximately one-fifth of patients. These findings underscore the importance of comprehensive complication reporting, careful patient selection, and prolonged clinical surveillance. In the absence of a comparative cohort and standardized severity assessment, definitive conclusions regarding the relative safety of this technique cannot be drawn.

The relationship between postoperative complications and time to full weight-bearing was also evaluated. Although the descriptive distribution suggested that some patients experiencing complications required longer periods before achieving unrestricted weight-bearing, the association was not statistically significant in the exact analysis. Given the small number of patients in several time categories, the findings remain imprecise and should be regarded as inconclusive rather than indicative of a statistically supported association.

Postoperative rehabilitation represents an important component of cosmetic femoral lengthening, particularly during the prolonged period of restricted weight-bearing required for regenerate consolidation. Although all patients followed a structured rehabilitation program, the potential effects of weight-bearing restrictions on muscle strength, joint mobility, gait recovery, and daily functioning were not systematically quantified. Furthermore, time to unrestricted weight-bearing was available, whereas the time required to achieve complete functional recovery was not documented using standardized outcome measures. Therefore, the rehabilitation findings should be interpreted as recovery milestones rather than evidence of complete functional restoration.

Several limitations of the present study should be acknowledged. First, its retrospective, single-center design and the absence of a comparative group limit causal inference and prevent direct comparisons with alternative limb-lengthening techniques. Second, detailed baseline anthropometric measurements, including femoral and tibial segmental lengths, were not systematically available, preventing a comprehensive assessment of postoperative lower-extremity proportional changes. Third, planned lengthening targets and certain treatment-related parameters were incompletely documented, limiting the assessment of target achievement and factors influencing the final length gain.

Fourth, standardized radiographic measures of regenerate quality, cortical bridging, and consolidation were not consistently available across the cohort. Fifth, detailed complication characteristics, including severity, timing, treatment, and long-term outcomes, were incompletely recorded in the available dataset. Sixth, standardized functional outcome measures, gait assessments, and validated psychological or patient-reported outcome instruments were not systematically collected. Although postoperative pain and time to full weight-bearing were documented, these parameters do not provide a comprehensive evaluation of functional recovery.

Finally, the relatively small sample size and limited number of patients with complications reduce the precision of subgroup analyses. These limitations emphasize the need for prospective studies incorporating standardized anthropometric, radiographic, functional, and patient-reported assessments.

Ongoing research remains essential to further refine the LON method, explore its long-term outcomes, and expand its clinical utility across broader patient populations.

## 5. Conclusions

In this retrospective cohort of 48 patients undergoing bilateral cosmetic femoral lengthening, the LON technique achieved a mean recorded length gain of 7.14 cm (range, 5.0–9.5 cm), with complications documented in 20.8% of patients. Patient age was weakly inversely associated with the magnitude of lengthening achieved, whereas no statistically significant association was identified between age and complication occurrence. Although these findings support the feasibility of LON-assisted cosmetic femoral lengthening in appropriately selected patients, larger prospective comparative studies incorporating standardized radiographic assessments and long-term functional outcomes are warranted to establish its safety and effectiveness.

## Figures and Tables

**Table 1:** Baseline demographic and clinical characteristics of the study cohort.

Characteristic	Value
Patients, n	48
Lengthened femora, n	96
Age, years, mean ± SD (range)	31.50 ± 9.43 (19–62)
Sex, male/female, n (%)	44 (91.7%)/4 (8.3%)
Preoperative height, cm, mean ± SD (range)	167.46 ± 6.05 (150.0–181.0)
Preoperative femoral segment length, cm	Not available in source dataset
Body mass index, kg/m^2^	Not available in source dataset
Planned femoral lengthening, cm	Not available in source dataset

**Table 2:** Achieved lengthening and postoperative recovery outcomes in 48 patients.

Outcome (Patient-Level Unless Specified)	Mean ± SD	Range
Recorded achieved lengthening, cm	7.14 ± 1.24	5.0–9.5
Height-based lengthening ratio, %	4.27 ± 0.73	2.86–5.57
Postoperative height, cm	174.71 ± 6.20	156.0–187.0
Time to full weight-bearing, months	5.88 ± 0.82	5–9
Recorded lengthening, complication group, cm	6.68 ± 1.17	5.0–8.5
Recorded lengthening, no-complication group, cm	7.27 ± 1.24	5.0–9.5
Not available		
Left femoral lengthening, cm	Not available	Not available
Elongation relative to baseline femoral length, %	Not calculable	Not calculable

## Data Availability

The datasets used and/or analysed during the current study are available from the corresponding author on reasonable request.

## Abbreviation

LON	Lengthening Over Nail
